# Genetics of Hearing Impairment

**DOI:** 10.3390/genes13050852

**Published:** 2022-05-11

**Authors:** Hannie Kremer, Ignacio del Castillo

**Affiliations:** 1 Hearing and Genes, Department of Otorhinolaryngology, Radboud University Medical Center, 6525 GA Nijmegen, The Netherlands; hannie.kremer@radboudumc.nl; 2 Department of Human Genetics, Radboud University Medical Center, 6525 GA Nijmegen, The Netherlands; 3 Donders Institute for Brain, Cognition and Behaviour, Radboud University Medical Center, 6525 GA Nijmegen, The Netherlands; 4 Servicio de Genética, Hospital Universitario Ramón y Cajal, Instituto Ramón y Cajal de Investigación Sanitaria (IRYCIS), 28034 Madrid, Spain; 5 Centro de Investigación Biomédica en Red de Enfermedades Raras (CIBERER), 28034 Madrid, Spain

The inner ear is a complex structure at the cellular and molecular levels. Many different genes and proteins play roles in the development and maintenance of the structure and its function through participating in diverse molecular networks. A defect in any of these components can result in hearing impairment. Consequently, hearing impairment encompasses a wide variety of disorders that are clinically and genetically heterogeneous. Understanding their genetic causes and their pathophysiological mechanisms and characterizing the resulting phenotypes are essential for developing novel therapies that target the specific defects. This Special Issue consists of 15 original research articles and 3 reviews that address different issues in the field of the genetics and molecular biology of hearing impairment, including genetic epidemiology, diagnostic strategies, genotype–phenotype correlations, pathophysiological mechanisms and murine models.

The importance of describing known as well as novel variants and the associated phenotypes in genes previously reported to be associated with hearing loss is often underestimated. In medical genetic practice, however, confirmation of disease association for genes and knowledge of genotype–phenotype correlations are highly relevant in the process of variant interpretation for the counseling of families and for patient management. In this Special Issue, Lachgar et al. report a truncating variant in *HOMER2*, which is only the third variant associated with hearing loss (DFNA68) [1]. All three variants affect the coiled-coil region of the HOMER2 protein and the phenotype in the corresponding families is similar, although variant-dependent variation in the severity of hearing loss might occur, but this needs to be confirmed. In Wonkam-Tingang et al., the second family is reported with hearing loss associated with compound heterozygous variants in *CLIC5* (DFNB103) [2]. In addition to supporting the association of *CLIC5* with hearing loss, the phenotype is also shown to be similar to that in the first family with non-syndromic prelingual sensorineural hearing loss, progressing to profound [3]. Vona et al. review the pathogenic genetic variants of *OTOF*, the gene encoding otoferlin, and their phenotypic consequences [4]. Otoferlin is located at the auditory ribbon synapse, where it plays a dual role as a calcium sensor in the exocytosis of synaptic vesicles and as a priming factor for fast vesicle replenishment. Consequently, otoferlin defects lead to an auditory synaptopathy. Over 200 pathogenic variants have been reported in *OTOF*, and most of them result in a prelingual, profound hearing impairment (HI). However, the phenotypic spectrum is broader than initially expected. Vona et al. pay special attention to reviewing less-common phenotypes, such as milder or progressive hearing losses, and the intriguing temperature-sensitive auditory synaptopathy. Challenges for clinical and genetic diagnosis are discussed, as well as their relevance for newborn hearing screening protocols and for the development of gene therapy clinical trials. In addition, *PJVK* defects have been described to underlie auditory neuropathy spectrum disorder (ANSD), but the gene has been associated with cochlear hearing loss as well. Domínguez-Ruiz et al. identified novel *PJVK* variants in a case with ANSD and both known and novel variants of the gene in cochlear hearing loss [5]. The authors provided an overview of all *PJVK* variants reported to underlie ANSD and/or cochlear hearing loss, which revealed that ANSD cases have at least one allele with a missense variant. Although this suggests that specific missense variants lead to ASND, the genotype–phenotype correlations are more complicated. This is further discussed in the article, as are insights into *PJVK* expression and function and the outcome of cochlear implants in patients with *PJVK* defects.

For genes that can cause syndromic as well as non-syndromic hearing loss when defective, it is even more important to understand the genotype–phenotype correlations. Two articles in this issue report families with non-syndromic hearing loss caused by missense variants in *CDH23* [6,7]. Three (novel) missense variants in this gene underlie non-syndromic hearing loss (DFNB12). All three affect the extracellular cadherin domains, and two of the variants are in the highly conserved Ca^2+^-binding domains. This confirms the previously observed association of bi-allelic missense variants with DFNB12 and not Usher syndrome type Id. The interpretation, and thus reporting, of variants in *CDH23* and other genes that are underlying both non-syndromic as well as Usher syndrome is a challenge in medical genetic practice and can lead to insecurity with parents about the future vision of their child. Also for defects of *GREB1L*, the phenotypic variability is high, as is typical for neurocristopathies. Schrauwen et al. describe two *GREB1L* variants in families with non-syndromic profound hearing loss [8]. In one of these families, temporal bone imaging revealed aplasia of the cochlea and of the cochlear nerve. A review of the literature, performed by the authors, indicated that in 14% of cases/families, dominantly inherited *GREB1L* disease is associated with an ear phenotype.

Two articles in this issue report novel cases with pathogenic variants in genes involved in Perrault syndrome, a disorder associating hearing loss with ovarian dysgenesis. Additionally, some patients develop neurological manifestations. Perrault syndrome is genetically heterogeneous, as eight genes are known to be involved. Zafar et al. report homozygous pathogenic variants in two of them, *CLPP* and *LARS2* [7]. These variants were found, respectively, in two Pakistani consanguineous familial cases with apparently non-syndromic HI. This is a common feature that illustrates the challenge of diagnosing this syndrome clinically. Indeed, male affected subjects, in the absence of neurological signs, only show HI. Moreover, ovarian dysgenesis cannot be detected in pre-pubertal affected females, and later, it is usually diagnosed after the second decade of life. Meanwhile, HI remains the only clinical sign. Also in this Special Issue, Oziębło et al. report two sisters with two novel compound heterozygous pathogenic variants, which confirm the involvement of *RMND1* in Perrault syndrome [9]. In addition to the classical features of the syndrome, a mild chronic kidney disease was observed in both sisters. Previously, mutations in *RMND1* had been reported to cause a more severe multiorgan phenotype, which includes neonatal lactic acidosis, encephalopathy, hearing loss and infantile-onset renal failure. Interestingly, a genotype–phenotype correlation is starting to emerge, so that missense variants (such as those reported by Oziębło et al.) would result in Perrault syndrome with mild kidney disease, whereas truncating variants may lead to the more severe phenotype. Identification and characterization of additional cases and mutations will show whether this hypothesis holds true.

Epidemiological studies provide useful data on which genes and causative genetic variants are more frequently involved in HI in each population. Accordingly, strategies for genetic diagnosis can be adapted to those particularities and to the resources and facilities of the different Services of Genetics. Three articles in this Special Issue report on epidemiological data for DFNB1, the most frequent type of non-syndromic HI. Resmerita et al. screened a cohort of 291 patients with congenital non-syndromic HI from Northeastern Romania, by using Multiplex-Ligation-dependent Probe Amplification (MLPA) followed by Sanger sequencing of the *GJB2* coding region [10]. Biallelic DFNB1 mutations were found in about 30% of the cases, the c.35delG variant being the most frequent (83% of pathogenic alleles), figures that are similar to those observed in other European populations [11]. As regards mutations outside the *GJB2* coding region, Resmerita et al. did find the splice-site variant c.-23+1G>A but not the large deletions that are more frequent in populations of Western Europe. A different DFNB1 landscape is observed in Argentina. Buonfiglio et al. screened a cohort of 600 Argentinean patients with non-syndromic HI by Sanger sequencing of the *GJB2* coding region and flanking sequences, and by PCR-detection of the two more common large deletions in the DFNB1 region [12]. Biallelic pathogenic variants were found in 36% of the familial cases and 15.5% of the sporadic cases. These different figures are a common feature in all tested populations, and illustrate the need to report data for familial and sporadic cases separately to allow for comparison with other studies. The most frequent variant was again c.35delG (52% of pathogenic alleles), and remarkably, the del(*GJB6*-D13S1830) and del(*GJB6*-D13S1854) large deletions accounted for over 8% of the pathogenic alleles. In the third article on DFNB1 in this Special Issue, Zytsar et al. demonstrate common founders and provide estimates of mutation ages for three *GJB2* pathogenic variants in Tuvinians and Altaians, two Turkish-speaking peoples from Southern Siberia [13]. A common founder explains the remarkably high frequency of the c.516G>C variant (up to 63% of pathogenic alleles in Tuvinians). Interestingly, this variant seems to be endemic in these populations, as it has not been reported elsewhere outside this region. Investigating the genetic causes of HI in isolated, less studied populations contributes to broadening our knowledge on the spectra of pathogenic variants and may lead to the identification of novel genes involved in these disorders.

The advent of massively parallel DNA sequencing (MPS) is boosting the studies on genetic epidemiology of HI, as it has solved the long-standing problem of screening a large number of genes in a cost-effective manner. Different screening strategies are being used. Morgan et al. investigated 125 Italian patients through a battery of techniques: Sanger sequencing of *GJB2* and *MTRNR1*, PCR-detection of DFNB1 large deletions, MLPA for deletions and duplications of *STRC* and *OTOA*, and whole-exome sequencing (WES) [14]. *GJB2* pathogenic variants accounted for 20% of the cases. Causative variants were found in an additional 26% of cases, in 24 different genes. In another study, García-García et al. used an MPS panel of 59 genes to investigate a cohort of 118 Spanish patients [15]. Causative variants were found in 40% of cases, in 19 different genes. In both studies, *GJB2* and *STRC* were the most frequently mutated genes among the recessive cases, and *MYO6* among the dominant ones. Finally, in the third broad epidemiological study in this Special Issue, Doll et al. investigated 21 Pakistani consanguineous families with autosomal recessive HI [16]. The cohort included 5 syndromic and 16 non-syndromic cases. The screening strategy combined autozygosity mapping with exome sequencing. Causative pathogenic variants were found in 13 families (62%), in 7 genes. In non-syndromic cases, the most frequently involved gene was *GJB2* (3 families). Pathogenic variants were also found in *MYO7A* (3 families) and *CDH23* (2 families), genes that are involved in non-syndromic HI as well as in Usher syndrome. Indeed, retinitis pigmentosa was present in only two of the *MYO7A* families. The results of these three studies show the diversity of pathogenic variants in different genes among populations. Broad studies on larger cohorts are needed in all populations to reveal the local and global epidemiological landscapes, whose knowledge is essential to orientate the strategies of genetic diagnosis and development of specific therapies.

In contrast to many of the studies in this Special Issue which address monogenic forms of non-syndromic hearing loss, the article by Escalera-Balsera et al. addresses a genetically more complex type of hearing loss, i.e., familial Meniere disease (FMD) (episodic vertigo associated with sensorineural hearing loss) [17]. In a systematic review of the literature, the authors found 20 rare variants in 11 genes to be (potentially) associated with FMD. They classified the variants for their potential deleterious effects and addressed population frequencies. Only a single candidate gene, *OTOG*, was reported to harbor potentially deleterious variants in more than a single family. The authors concluded that associations of genes with FMD need to be replicated in order to determine the causative effect of variants in these candidate genes.

Mice have proven to be excellent models for studying the function and pathophysiology of genes associated with hearing loss in humans, but there are exceptions to this. Tona et al. identified compound heterozygous *TBC1D24* variants in a Pakistani family with intrafamilial phenotypic heterogeneity [18]. Affected family members either suffered from non-syndromic hearing loss or hearing loss and seizures. The authors set out to model *TBC1D24*-associated disease in mice. Although the seizure phenotype was recapitulated in mice with compound heterozygous truncating variants of this gene, none of the models displayed a hearing loss phenotype. This might be explained by differences in the cochlear expression of *TBC1D24/Tbc1d24* in humans and mice. The authors address and discuss additional potential explanations for the phenotypic differences between mice and humans with *Tbc1d24/TBC1D24* defects. For one of the variants, molecular dynamic simulations of peptide structure pointed towards such an explanation.

Perrino et al. employed the mouse to model the potential role of *USH2A* defects in central auditory processing disorder (CAPD), as was indicated in a genome-wide association study (GWAS) [19]. The authors indeed obtained indications for an effect of *Ush2a* defects on the structure of the central auditory system, both in homozygous knockout as well as heterozygous knockout mice. This suggest that cochlear development altered by *USH2A* defects can lead to a secondary effect on the brain regions that function in auditory processing.

Knowledge on the cellular mechanisms that lead to the different types of genetic HI is essential to develop specific therapies. Hayashi et al. reviewed the insights in autophagy in inner ear development and maintenance [20]. These insights are most extensive for hair cells, auditory neurons, and brain stem nuclei. The authors also highlighted the involvement of autophagy in hereditary hearing loss, more specifically for DFNA5 (*GSDME*) and DFNA59 (*PJVK*). Autophagy is essential for cell fate by controlling the balance between cell survival and cell death in conditions of cellular stress. Therefore, the autophagy pathway is an interesting target for therapeutic intervention in hearing loss. One could also hypothesize that variants in genes functioning in autophagy might be modifying factors in dominantly inherited types of hearing loss which display large intrafamilial variability and in which toxic gain of function effects of mutant proteins are often indicated.

The articles and reviews in this Special Issue are representative of the many research lines that are currently active in the field of inherited hearing impairment. These efforts are providing essential data for the comprehension of these highly heterogeneous disorders and for the development of specific new therapies, whose application to humans looks closer than ever.

## References

[1] Lachgar M., Morín M., Villamar M., del Castillo I., Moreno-Pelayo M.Á. (2021). A Novel Truncating Mutation in HOMER2 Causes Nonsyndromic Progressive DFNA68 Hearing Loss in a Spanish Family. Genes.

[2] Wonkam-Tingang E., Schrauwen I., Esoh K.K., Bharadwaj T., Nouel-Saied L.M., Acharya A., Nasir A., Adadey S.M., Mowla S., Leal S.M. (2020). Bi-Allelic Novel Variants in CLIC5 Identified in a Cameroonian Multiplex Family with Non-Syndromic Hearing Impairment. Genes.

[3] Zazo Seco C., Oonk A.M., Domínguez-Ruiz M., Draaisma J.M., Gandía M., Oostrik J., Neveling K., Kunst H.P., Hoefsloot L.H., del Castillo I. (2015). Progressive hearing loss and vestibular dysfunction caused by a homozygous nonsense mutation in CLIC5. Eur. J. Hum. Genet..

[4] Vona B., Rad A., Reisinger E. (2020). The Many Faces of DFNB9: Relating OTOF Variants to Hearing Impairment. Genes.

[5] Domínguez-Ruiz M., Rodríguez-Ballesteros M., Gandía M., Gómez-Rosas E., Villamar M., Scimemi P., Mancini P., Rendtorff N.D., Moreno-Pelayo M.A., Tranebjaerg L. (2022). Novel Pathogenic Variants in PJVK, the Gene Encoding Pejvakin, in Subjects with Autosomal Recessive Non-Syndromic Hearing Impairment and Auditory Neuropathy Spectrum Disorder. Genes.

[6] Ramzan K., Al-Numair N.S., Al-Ageel S., Elbaik L., Sakati N., Al-Hazzaa S.A.F., Al-Owain M., Imtiaz F. (2020). Identification of Novel CDH23 Variants Causing Moderate to Profound Progressive Nonsyndromic Hearing Loss. Genes.

[7] Zafar S., Shahzad M., Ishaq R., Yousaf A., Shaikh R.S., Akram J., Ahmed Z.M., Riazuddin S. (2020). Novel Mutations in CLPP, LARS2, CDH23, and COL4A5 Identified in Familial Cases of Prelingual Hearing Loss. Genes.

[8] Schrauwen I., Liaqat K., Schatteman I., Bharadwaj T., Nasir A., Acharya A., Ahmad W., Van Camp G., Leal S.M. (2020). Autosomal Dominantly Inherited GREB1L Variants in Individuals with Profound Sensorineural Hearing Impairment. Genes.

[9] Oziębło D., Pazik J., Stępniak I., Skarżyński H., Ołdak M. (2020). Two Novel Pathogenic Variants Confirm RMND1 Causative Role in Perrault Syndrome with Renal Involvement. Genes.

[10] Resmerita I., Cozma R.S., Popescu R., Radulescu L.M., Panzaru M.C., Butnariu L.I., Caba L., Ilie O.D., Gavril E.C., Gorduza E.V. (2020). Genetics of Hearing Impairment in North-Eastern Romania-A Cost-Effective Improved Diagnosis and Literature Review. Genes.

[11] del Castillo I., Morín M., Domínguez-Ruiz M., Moreno-Pelayo M.A. (2022). Genetic etiology of non-syndromic hearing loss in Europe. Hum. Genet..

[12] Buonfiglio P., Bruque C.D., Luce L., Giliberto F., Lotersztein V., Menazzi S., Paoli B., Elgoyhen A.B., Dalamón V. (2020). GJB2 and GJB6 Genetic Variant Curation in an Argentinean Non-Syndromic Hearing-Impaired Cohort. Genes.

[13] Zytsar M.V., Bady-Khoo M.S., Danilchenko V.Y., Maslova E.A., Barashkov N.A., Morozov I.V., Bondar A.A., Posukh O.L. (2020). High Rates of Three Common GJB2 Mutations c.516G>C, c.-23+1G>A, c.235delC in Deaf Patients from Southern Siberia Are Due to the Founder Effect. Genes.

[14] Morgan A., Lenarduzzi S., Spedicati B., Cattaruzzi E., Murru F.M., Pelliccione G., Mazzà D., Zollino M., Graziano C., Ambrosetti U. (2020). Lights and Shadows in the Genetics of Syndromic and Non-Syndromic Hearing Loss in the Italian Population. Genes.

[15] García-García G., Berzal-Serrano A., García-Díaz P., Villanova-Aparisi R., Juárez-Rodríguez S., de Paula-Vernetta C., Cavallé-Garrido L., Jaijo T., Armengot-Carceller M., Millán J.M. (2020). Improving the Management of Patients with Hearing Loss by the Implementation of an NGS Panel in Clinical Practice. Genes.

[16] Doll J., Vona B., Schnapp L., Rüschendorf F., Khan I., Khan S., Muhammad N., Alam Khan S., Nawaz H., Khan A. (2020). Genetic Spectrum of Syndromic and Non-Syndromic Hearing Loss in Pakistani Families. Genes.

[17] Escalera-Balsera A., Roman-Naranjo P., Lopez-Escamez J.A. (2020). Systematic Review of Sequencing Studies and Gene Expression Profiling in Familial Meniere Disease. Genes.

[18] Tona R., Lopez I.A., Fenollar-Ferrer C., Faridi R., Anselmi C., Khan A.A., Shahzad M., Morell R.J., Gu S., Hoa M. (2020). Mouse Models of Human Pathogenic Variants of TBC1D24 Associated with Non-Syndromic Deafness DFNB86 and DFNA65 and Syndromes Involving Deafness. Genes.

[19] Perrino P.A., Newbury D.F., Fitch R.H. (2021). Peripheral Anomalies in USH2A Cause Central Auditory Anomalies in a Mouse Model of Usher Syndrome and CAPD. Genes.

[20] Hayashi K., Suzuki Y., Fujimoto C., Kanzaki S. (2020). Molecular Mechanisms and Biological Functions of Autophagy for Genetics of Hearing Impairment. Genes.

